# Giant cell tumor of bone: Is curettage the answer?

**DOI:** 10.4103/0019-5413.32040

**Published:** 2007

**Authors:** Shishir Rastogi, I Prashanth, Shah Alam Khan, Vivek Trikha, Ravi Mittal

**Affiliations:** Department of Orthopaedics, All India Institute of Medical Sciences, New Delhi - 110029, India

**Keywords:** Adjuvant therapy, extended curettage, giant cell tumor

## Abstract

**Background:**

Giant cell tumors (GCT) are neoplasms of mesenchymal stromal cells with varied manifestations. There is no uniform accepted treatment protocol for these tumors,

**Materials and Methods:**

49 cases of proven giant cell tumors of appendicular skeleton, 27 prospective and 22 retrospective constituteed this study. The retrospective cases were collected by using computerized data base collection method. The patients were evaluated clinically, radiologically and by histology. Companacci grading and Enneking staging was used in the study. Two treatment modalities were used a) extended curettage (with/ without bone grafting/ cementation) or b) wide excision and reconstruction with a prosthesis or arthrodesis. Functional evaluation was done by Enneking's system. Chi square tests, mann-whitney test and ANOVA were used for statistical analysis.

**Results:**

The average age was 26.82 years (16-50 years). 25 patients (51%) were recurrent GCT at presentation. The commonest site was lower end of femur (16 cases, 32.65%) and upper end of tibia (13 cases, 26.53%). 40 (81.63%) tumors had less than 5 mm of subchondral bone free of tumor. 35 (71.43%) tumors were Enneking's surgical stage III and companacci grade III. Pathological fractures were seen in 12 (24.49%) cases. Intra-lesional currettage was used in 28 and enbloc excision in 19 patients and 2 (4.08%) underwent amputation. The average follow up period was 18.6 months (range 2-84). One recurrence was seen in a grade III recurrent distal radial lesion in the intralesional curettage group (3.57%) Enneking's functional score with intralesional curettage (25.41) was better than enbloc excision (21.37). Enbloc excision had higher rates of infections (36.84 % Vs 25%) and soft tissue coverage problems (21.05% Vs 0).

**Conclusion:**

Intralesional therapy has a better functional outcome and less complications than enbloc excision, albeit with a high recurrence rate which can however be effectively treated with repeat extended curettage.

## INTRODUCTION

Giant cell tumors (GCT) of bones have been described histologically as neoplasms of undifferentiated mesenchymal stromal cells with the presence of abundant giant cells; radiographically presenting as an eccentric lytic lesion at the ends of long bones and clinically as a benign but often locally aggressive lesion. It has a tendency towards local recurrence and occasionally malignant change.

Bloodgood,^1^ in 1912, coined the term giant cell tumor and emphasized the benign nature of this tumor. Modern view of GCT began in 1940 when Jaffe and associates proved these tumors as a benign aggressive.^2^,^3^ This terminology is misleading, because 3% of giant cell tumors are primarily malignant or will undergo malignant transformation and metastasize.^2^

The treatment of GCT has changed from amputation at the beginning of century to curettage and excision. The current suggestions have varied from curettage for all lesions^1^,^3^ to wide excision for each tumor.^4^ Increased recurrence rates seen with curettage alone led many to use adjuvants. This study was conducted on all the patients with appendicular lesions treated since 2000, comparing intralesional curettage with wide excision, for recurrence, complications and residual limb function.

## MATERIALS AND METHODS

Ours was a combined retrospective-prospective study. Patients treated between January 2000 to January 2006 were included in the study. Patients treated from January 2000 to January 2004 were studied retrospectively from previous hospital records and followed up regularly at regular intervals. Cases from January 2004 to January 2006 were studied prospectively and followed up regularly. A total of 49 patients were studied, with 27 in the prospective and 22 in the retrospective group. Average follow up period was18.6 months (range 2-84 months).

Only biopsy proven giant cell tumors of appendicular skeleton were included in the study. Spinal lesions were excluded from study. Evaluation of patients in retrospective group was done by available hospital records using a computerized database. Patients registered during the course of the study were evaluated with clinical examination, radiological evaluation and histopathology. All data was recorded on a prefixed proforma. Radiographs and MR scans were done for all patients. Chest radiographs were done in all patients. Site of lesion, (epiphyseal, epiphyseo-metaphyseal, metaphyseal or diaphyseal) was carefully evaluated. Size of radiolucent area were recorded as occupying less than one half, one-half and more than one-half diameter of bone in A-P view. Thickness of the subchondral bone at adjacent articular surfaces was measured radiologically and recorded as more than 5 mm, 5 mm or less or zero.^1^ Campanacci^5^ grading was used for cortical breach. Grade I tumor had a well marginated border of a thin rim of mature bone and the cortex was intact or slightly thinned but not deformed. Grade II tumor had relatively well defined margins but no radio-opaque rim. Grade III tumors had fuzzy borders.

Enneking^6^ staging was used preoperatively. Stage I is defined as a latent (inactive) lesion that is asymptomatic, intracompartmental and histologically benign. Stage II has been defined as active, symptomatic and intracompartmental. Stage III is an aggressive lesion that is extra compartmental. Recurrent lesions were compared with primary lesions for any radiographic aggressiveness like Campanacci grade and size on A-P radiograph. Recurrence was considered to be present when there was progressive lysis of more than 5 mm at cement-bone or graft-host interface or if there was an absence of a sclerotic rim at the above said interface.^7^ Peripheral calcification around a soft tissue mass of uniform density was the criteria for recurrence in soft tissues.^8^

**Figure 1:**
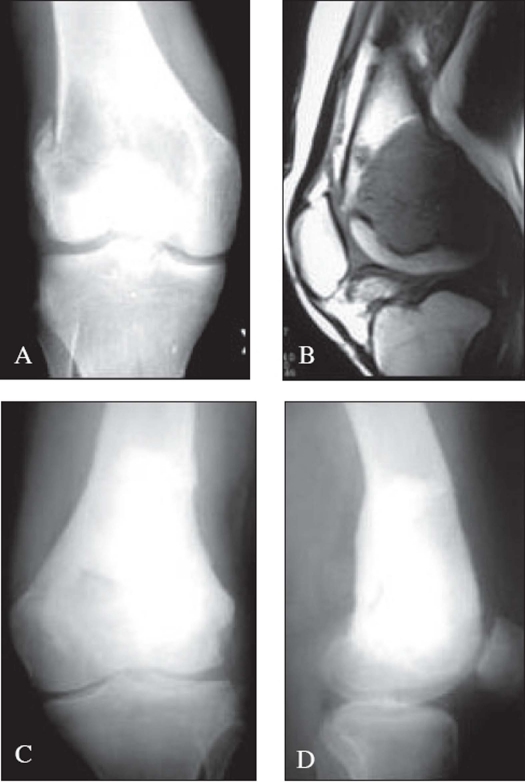
A) X-rays (A.P.) of the knee in a 17-year-old male with GCT of the distal femur with pathological fracture of the lateral femoral condyle. B) MRI of the same patient showing the pathological fracture and articular cartilage free of tumor. C), D). 7 month post operative x-rays (A.P. and lateral) of the same patient treated with curettage, bone graft and bone cement

Two treatment modalities were used: Extended curettage (with/without bone grafting/cementation) or wide excision and reconstruction with a prosthesis or arthrodesis. The type of reconstruction was decided by a affordability of the patient for prosthesis. Extended curettage was [Figure 1] done when atleast 2 mm of subarticular bone was free of the tumor with no soft tissue spillage as assessed on a recent MRI. Extended curettage was done using a high speed (70000 rpm, Midasrex^©^) burr. Phenol (1 ml of melted phenol mixed with 10 ml of normal saline) was used as chemical adjuvant in all cases. The subchondral region of cavity was then packed with autogenous bone graft obtained from iliac crest. Either fibular cortical autograft or irradiated corticocancellous allograft bone obtained from bone bank was used. Polymethylmethacrylate (PMMA) was also used in some cases when the size of the intact articular surface after extended curettage needed additional support along with bone grafts. This was to prevent prolonged immobilization. When necessary, standard internal fixation was used. Wide excusion was done when the tumor as assessed by recent X-rays and MR scane had extended to within 2 mm of articular cartilage or when it it had breached the cortex to extend to surrounding soft tissues. Post-operatively, patients were followed up at weekly intervals in first month, fortnightly for next 2 months and monthly thereafter. X-rays were taken at every visit after the eight weeks and then every six weeks. Functional evaluation was done by Enneking's system. This system is applicable in evaluating limb salvage surgeries. This evaluates pain, function and emotional acceptance, besides dexterity as a measure of upper limb functions and walking ability, gait as a measure of lower limb functions. It was done 6 months post surgery or in the last follow up in patients with less than 6 months followup (2 cases). Chi square tests, Mann-Whitney test and ANOVA were used for statistical analysis.

## RESULTS

A total of 49 cases, 22 (44.89%) cases from retrospective group and 27 (55.10%) patients from the prospective group were studied and followed up. The age of the patient at presentation varied from 16 to 50 years. 28 cases (57.14%) were in 3^rd^ decade of life. The male: female ratio was 1.5:1. The commonest site was distal femur in 16 cases (32.65%), followed closely by upper end tibia in 13 cases (26.53%). Eight cases (16.33%) were seen in distal end radius, while 4 cases (8.16%) each were seen in lower end tibia and proximal femur. Lesions were also seen in proximal humerus (2 cases, 4.08%), lower end humerus (1 case, 2.04%) and 2^nd^ metacarpal (1 case, 2.04%) Lower limbs accounted for 37 cases (75.51%) while 12 cases (24.49%) involved upper extremity. 25 (51%) of our patients had recurrence on presentation, while the other 24 (49%) were primary. The initial treatment details of recurrent cases were not available accurately in all cases as they were operated in other hospitals. Epiphyseo-metaphyseal was the commonest location in 44 (89.8%) cases. In 39 cases (79.6%) tumors occupied more than half the width of bone on A-P radiographs, while the rest 10 (20.4%) had sizes less than half width of bone. 30 lesions (61.22%) had subchondral tumor free bone of less than 5 mm, while in 11 lesions (22.45%) the tumor had extended right upto articular cartilage. Eight lesions (16.33%) were far away from articular cartilage with more than 5 mm of uninvolved subchondral bone.

Out of 49 patients, 1 was Campanacci grade I (2.04%), 13 were grade II (26.53%) and 35 were grade III (71.43%). Out of 49 patients, 1 was of Enneking stage I (2.04%), 13 were of stage II (26.53%) and 35 (71.43%) were of stage III. There was no statistically significant difference (*P*=0.208) in Campanacci grade or Enneking stage, between the primary or recurrent lesions.

6 of 24 (25%) primary lesions and 3 of 25 (12%) recurrent lesions presented with size less than half the width on A-P radiograph. 18 of 24 (75%) primary and 22 of 25 (88%) recurrent lesions had more than half the width on A-P radiograph. Hence, recurrent lesions, when compared to primary lesions did not have larger sizes at presentation (*P*=0.404). This may be due to late presentation of primary cases to our clinic. Also, recurrent lesions, when compared to primaries did not have any difference in subchondral tumor free zone.

Pathological fractures were seen in 12 patients (24.49%) commonly (5/12, 41.67%) in lower end femur. It was also seen in lower end radius (3/12, 25%), upper end femur (2/12, 16.67%), upper end tibia (1/12, 8.33%) and upper end humerus (1/12, 8.33%). Out of 12 patients with pathological fractures, 6 (50%) were primary and 6 (50%) were recurrent lesions; pathological fractures were not commoner in recurrent lesions (*P*=0.935). The mean follow up score in patients with pathological fractures was 22.58, while in those without fracture was 23.76. This was statistically not significant (*P*=0.564). One out of 12 patients (8.33%) with pathological fracture had recurrence on follow up which however was not significant (*P*=0.076).

Out of 49 patients, 28 (57.14%) were treated with curettage. After curettage, bone grafting alone was used in 23 (82.14%) patients, while one patient, a fourth recurrence in proximal tibia was treated with only bone cement. Four patients were treated with both bone grafting and bone cement after curettage [Figure 1], two of them receiving morcellized allograft in addition to autologous bone cement and bone graft. Bone cement was used where it was deemed necessary to provide additional structural support to articular cartilage, in addition to bone grafting. No structural allograft were used in our study.

**Figure 2:**
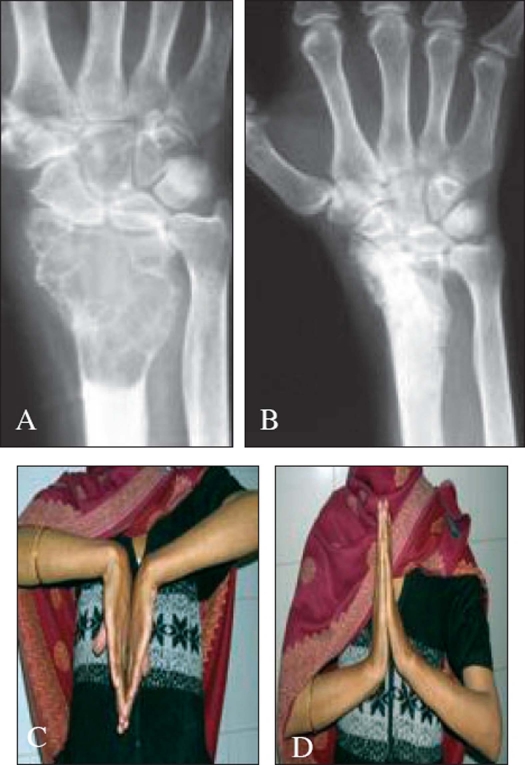
A) Pre-operative x-ray of recurrent GCT of distal end radius in a 35-year -old female. B) 8 month post-operative x-ray of the same patient. C), D) Clinical picture demonstrating good range of wrist movements in the same patient at 8 months follow up

Nineteen (38.77%) patients were treated with wide excision and 2 (4.08%) underwent amputation. Out of 19 patients 13 had GCT around the knee joint. Out of which 7 were treated with arthrodesis, six with prosthesis. Four lesions of distal radius were [Figure 2] excised and non vascularized fibular grafting was done. One patient with GCT in lateral condyle of humerus was treated with excision and replacement with fibular head. One recurrent lesion of 2^nd^ metacarpal underwent vascularized toe transfer.

Campanacci grades I (1/1, 100%) and II (12/13, 92.31%) have been predominantly treated with curettage while grade III tumors were treated predominantly with wide excision (18/35, 51.43%) [Table 1]. Two underwent amputation while rest of grade III lesions (15 cases, 42.86%) were curetted. Similarly, Enneking stages I (1/1, 100%) and II (12/13, 92.31%) have been predominantly treated with curettage. Enneking stage III tumors have undergone predominantly wide excision (18/35 o, 51.43%). Two (5.71%) of stage III lesions were amputated, while 15(42.86%) underwent curettage. Out of 12 patients with pathological fractures, 5 (41.67%) were treated with curettage while the other 7 (58.33%) underwent wide excision. Internal fixation was used in only 2 out of these 12 patients who underwent arthrodesis.

**Table 1:** Treatment modalities for the three campanacci grades

Campanacci grade	Treatment		Total
		
	Wide excision	Curettage + BG	
I		1	1
II	1	12	13
III	18	15	33
Total	19	28	47*

* 2 pts. were amputated

Seventeen of 24 primary cases were treated with curettage and bone grafting (70.83%), while 7 underwent wide excision (29.17%). 11 of 25 recurrent lesions were treated with repeat curettage (44%) and 12 underwent wide excision (48%). Both patients who underwent amputation were recurrent lesions (8%). One patient was amputated at above knee level for recalcitrant infection, while the other was disarticulated at hip for extensive local spread.

Out of 14 patients with Campanacci grade I or II, none recurred. One (2.86%) patient out of 35 grade III GCTs recurred. None of Enneking stage I or II recurred. One recurrence in 35 stage III patients (2.86%) was noted. Recurrence was seen in 1 patient (2.04%), who had undergone curettage for a recurrent lesion in distal radius and had fracture at presentation. No recurrences were noted with wide excision [Table 2].

**Table 2:** Recurrence rates in the two treatment groups (curettage and wide excision)

Treatment modality	Recurrence		Total
		
	Absent	Present	
Wide excision	19	-	19
Curettage + BG	27	1	28
Total	47*	1	47

BG = Bone grafting,

* 2 pts. were amputated

**Figure 3:**
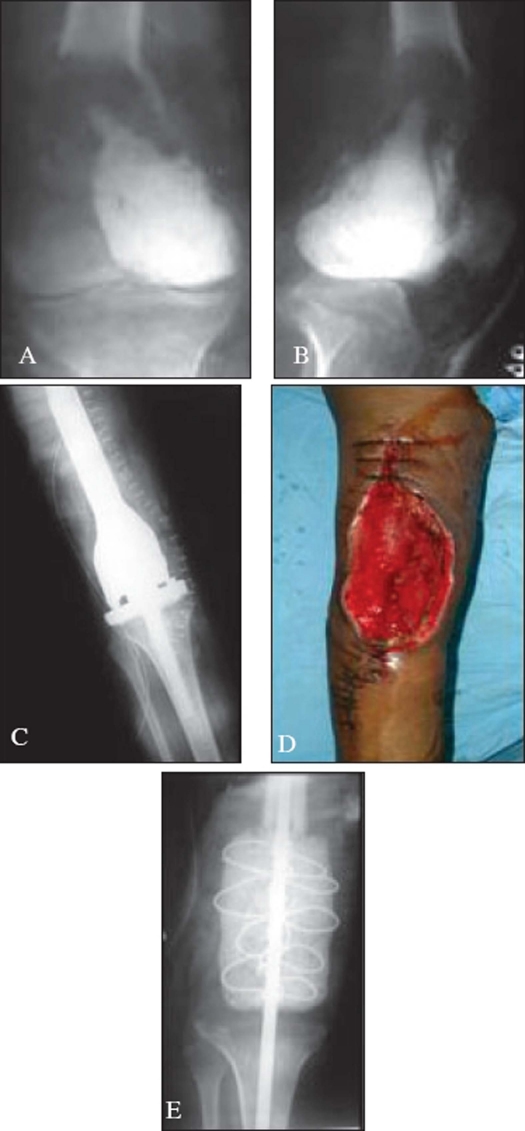
A), B) X-rays (A.P.) and lateral) of knee shows a recurrent GCT distal end femur in a 25-year-old female. C) Postoperative x-ray of the same patient showing tumor excision and endoprosthesis insertion. D) Post operative picture of the same patient with coverage problems of the wound. E) 5 month post op picture of the same patient showing removal of prosthesis and cement spacer insertion. Patient underwent ampution after 13 months due to uncontrolled infection

The average follow up was 18.6 months with a median of 19 months. Six patients in curettage group and 5 patients in wide excision group had a follow up of less than 12 months. The average functional score of 47 patients was 23.49. In 28 patients with curettage and bone grafting it was 25.41 while with wide excision it was 21.37, this difference being significant (*P*=0.017). Three patients following wide excision, with complications like exposed plate due to soft tissue coverage problems and recurrent infections had poor scores [Figure 3]. Two underwent amputation for whom functional score cannot be applied. All complications were more frequent with wide excision. Wound infection was seen in 7 cases (25%) treated with curettage, which was low compared to 7 cases (36.84%) with wide excision. Soft tissue coverage problems were seen in 4 cases (21.05%) of wide excision group and none with curettage group (*P*=0.013). Other complications like carpal impingement etc. were seen more frequently in patients of distal end radius treated with wide excision (31.58%).

## DISCUSSION

Lack of a definite treatment protocol in treating GCT has resulted from a highly variable presentation of this ubiquitous bone lesion. Lack of studies from South East Asia has further compounded the problem with a deficient data on outcome of GCTs in Asian population.

The mean age of presentation was 26.8 yrs (16-50). Presentation was commonest in third decade. This is in accordance with previous studies.^1^,^2^,^5^,^10^ Male-female ratio in our study was 1.5:1. Campanacci reported an equal sex ratio for GCT.^5^ We found 29 (59.18%) of our lesions around the knee joint with 16 (32.65%) cases in distal end femur and 13 (26.53%) in upper end of tibia. Prognosis of GCT around knee joint is vital from a functional point of view. This has been shown by other authors as well.^2^,^10^,^11^

Pathological fracture was seen in 12 (24.49%) of our patients, commonly in lower end of femur (5 cases or 41.67%). Similar observations were made by Campanacci^5^ and Turcotte.[11] It's presence, however did not affect the final functional outcome in our study (*P*=0.564). Fifty one percent of our patients presented to us with first recurrence, following a primary procedure done outside. Recurrence was considered to be present when there was progressive lysis of more than 5 mm at cement-bone or graft-host interface or if there was an absence of a sclerotic rim at the above said interface.^7^ Lysis on host-graft interface can be analysed as graft has a different radiodensity than host bone until it is fully incorporated, when its density merges with that of host bone. Recurrent lesions did not have any greater tendency for fractures compared to primary tumors (*P*=0.935).

According to Schajowicz,^12^ curettage alone is an inadequate oncologic procedure for GCT but associated with better functional outcome compared to enbloc excision. Treatment is a balance between oncological adequacy and functional utility of the limb. Extended curettage with bone grafting was done in 57.14% of our patients, this being the commonest modality of treatment in the series. This treatment modality has been shown to be effective by other authors.^1^,^9^,^11^,^13^ 42.86% underwent wide excision. Curettage with bone grafting was the commonest modality in primary cases (70.83%) while wide resection was the commonest treatment for recurrent lesions (48%). Pathological fracture was not a contraindication to curettage and bone grafting in this study as was opined by Dreinhofer.^14^

Curettage in GCT is usually followed by adjuvant therapy either to achieve a more thorough tumor kill or to provide enhanced structural support around the joint. We used phenol with curettage. Phenol is known to cause protein coagulation, damages deoxyribonucleic acid and causes necrosis.^15^ Good results using phenol has been shown by other authors. Polymethylmethacrylate, cortico-cancellous autograft and allograft, Pamidronate, etc. have all been effectively used following curettage in GCT.^15^,^16^ Our experience is based on use of PMMA, phenol and bone grafts, with good functional outcome and low recurrence rates. We had only one recurrence among 28 patients with this procedure. Literature shows a variable recurrence rate of 4.5% to 52%.^1^,^3^,^5^,^10^,^11^ Meticulous pre-operative evaluation including staging and surgical procedure (previous scar incorporation in the incision, clean dissection, large cortical window, the use of high speed burr and use of adjuvants like phenol, cement^17^ can reduce recurrence rates effectively. The use of mechanical burr and process of exteriorization is known to reduce recurrence rate.^18^ On the contrary, several studies have ruled out any advantage of using an adjuvant.^19^,^20^ Extended follow up may well be required before evaluating success of the strategies used.

The mean follow up score at the last follow up was 23.5. Curettage had better functional outcome when compared to wide excision (*P*=0.017). Joint salvage is known to improve function. Wide excision was associated with more complication rates than curettage.

## CONCLUSION

The extended curettage is associated with a better functional outcome than wide excision. This is particularly important in the Indian context as most of our patients if given a choice, opt for curettage over an endoprosthetic replacement in view of financial constraints, functional limitations (particularly squatting) and more loss of productive working hours. Thus for Indian patients extended curettage with bone grafting in giant cell tumors is a cost-effective, easy technology option.
